# Supplementary material for: cGMP Signaling in Arterioles Revealed by FRET‐Based Real‐Time Measurement in Mice In Vivo
Source: Acta Physiol (Oxf). 2026 Sep 27;242(11):e70317. doi: 10.1111/apha.70317 (PMC13616743; doi:10.1111/apha.70317)
Supplement: Supplementary file 1 [file APHA-242-e70317-s001.docx]

**Supplementary Online Material**

**cGMP signaling in arterioles revealed
by FRET-based real-time measurement in mice in vivo**

Lukas Thiele^1,2^, Kjestine Schmidt^1,2^, Martin Thunemann^3,5^, Andreas Friebe^4^, Susanne Feil^3^, Robert Feil^3^, Cor de Wit^1,2^

1 Institut für Physiologie, Universität zu Lübeck, Lübeck, Germany

2 German Centre for Cardiovascular Research (DZHK), partner site North, Lübeck, Germany

3 Interfakultäres Institut für Biochemie, Universität Tübingen, Tübingen, Germany

4 Physiologisches Institut, Julius-Maximilians Universität, Würzburg, Germany

5 Current address: Department of Biomedical Engineering, Boston University, Boston, MA, USA

Address for correspondence:

Cor de Wit

Institut für Physiologie

Universität zu Lübeck

Ratzeburger Allee 160

23562 Lübeck, Germany

Telephone	+49-451-3101 7310

E-mail	dewit@uni-luebeck.de
ORCID-ID: 0000-0003-4621-0399

Running title: cGMP signaling in arterioles in vivo

**Figure S1**

**Fig. S1: Baseline ratio values before application of NO-donors**

Baseline ratio values are depicted as absolute numbers for the experiments plotted in figure 2 of the manuscript. The absolute ratio values were used as denominators to calculate the percent changes of the ratio shown in figure 2. The absolute baseline values were not different for the NO-donors (DEA/NO, SNAP, or SNP) at different concentrations (shown on the x-axis). Values are plotted as box-plots with the whiskers representing 1.5x the interquartile range. Ratio values are lower than in Fig. S2 due to the light source used for excitation in this experimental series (polychromator system).

**Figure S2**

**Fig. S2: Baseline ratio values before application of NO-donors during different treatments**

Baseline ratio values are depicted as absolute numbers for the experiments plotted in figures 3, 4B, and 4D of the manuscript. The absolute ratio values were used as denominators to calculate the percent changes of the ratio as shown in manuscript figures. Control values (white bars) are plotted next to values obtained during treatment (grey bars) for each NO-donor separately, baseline values before application of the different concentrations of each NO-donor are merged. A: ODQ decreased absolute baseline values before DEA/NO, but did not reduce baseline values before SNAP. B: IBMX or sildenafil (Sil) enhanced baseline values before application of SNAP, but not before DEA/NO. Values are plotted as box-plots with the whiskers representing 1.5x the interquartile range. *: *P*<0.05 vs. control (paired t-test). Ratio values are higher than in Fig. S1 due to the light source used for excitation in these experimental series (light-emitting diode).

**Figure S3**

**Fig. S3: Absolute ratio changes after application of NO-donors before and during PDE inhibition**

Ratio changes are depicted as absolute values calculated by subtracting the ratio before NO-donor application from the mean value measured during NO-donor application for the experiments plotted in figures 4B, 4C, and 4D of the manuscript. The absolute ratio changes induced by DEA/NO or SNAP were not enhanced in the presence of PDE inhibitors which replicates the results shown in figures 4B, 4C, and 4D of the manuscript. A: Control responses (DEA/NO, SNAP) were examined firstly, followed by IBMX. B: IBMX was applied first, followed by its washout (SNAP). C: Sildenafil (Sil) was examined only for the NO-donor SNAP. ns indicates no significant difference, * indicates *P*<0.05.
